# Routine and interval detection of locoregional breast cancer recurrences and risk of subsequent distant metastasis

**DOI:** 10.1007/s10549-022-06757-3

**Published:** 2022-10-31

**Authors:** Anouk H. Eijkelboom, Linda de Munck, Maaike de Vries, Anne Brecht Francken, Mathijs P. Hendriks, Luc Strobbe, Annemieke Witteveen, Marissa C. van Maaren, Sabine Siesling

**Affiliations:** 1 Department of Research and Development, Netherlands Comprehensive Cancer Organisation (IKNL), Utrecht, The Netherlands; 2 Department of Health Technology and Services Research, Technical Medical Centre, University of Twente, Enschede, The Netherlands; 3 Department of Surgical Oncology, Isala Clinics, Zwolle, The Netherlands; 4 Department of Medical Oncology, Northwest Clinics, Alkmaar, The Netherlands; 5 Department of Surgical Oncology, Canisius Wilhelmina Hospital, Nijmegen, The Netherlands; 6 Department of Biomedical Signals and Systems, Technical Medical Centre, University of Twente, Enschede, The Netherlands

**Keywords:** Breast cancer, Follow-up, Recurrence, Distant metastasis, Tumor characteristics

## Abstract

**Purpose:**

Follow-up for breast cancer survivors consists of after care and surveillance. The benefits of routine surveillance visits remain debatable. In this study we compared the severity of locoregional recurrences (LRRs) and the subsequent risk of a distant metastasis (DM) between LRRs detected at routine and interval visits.

**Methods:**

Women diagnosed with early breast cancer between 2003 and 2008 in one of the 15 participating hospitals, and who developed a LRR as first event after primary treatment, were selected from the Netherlands Cancer Registry (Cohort A). Chi-squared tests were used to compare the severity of routine- and interval-detected local recurrences (LRs) and regional recurrences (RRs), using tumor size, tumor grade, and number of positive lymph nodes. Data on the development of a subsequent DM after a LRR were available for a subset of patients (Cohort B). Cohort B was used to estimate the association between way of LRR-detection and risk of a DM.

**Results:**

Cohort A consisted of 109 routine- and 113 interval-LRR patients. The severity of routine-detected LRs or RRs and interval-detected LRs or RRs did not significantly differ. Cohort B consisted of 66 routine- and 61 interval-LRR patients. Sixteen routine- (24%) and 17 (28%) interval-LRR patients developed a DM. After adjustment, way of LRR-detection was not significantly associated with the risk of a DM (hazard ratio: 1.22; 95% confidence interval: 0.49–3.06).

**Conclusion:**

The current study showed that routine visits did not lead to less severe LRRs and did not decrease the risk of a subsequent DM.

**Supplementary Information:**

The online version contains supplementary material available at 10.1007/s10549-022-06757-3.

## Introduction

Breast cancer is the most prevalent type of cancer among women worldwide [1]. Due to its earlier detection and improved treatment, survival has increased significantly over the last couple of decades [2], leading to an increased number of patients receiving follow-up after curative treatment. Follow-up consists of aftercare and surveillance. Aftercare aims to signal, guide, and treat psychosocial and physical consequences of breast cancer (treatment), while surveillance aims to detect locoregional recurrences (LRRs) and second primary breast cancers (SPBCs) in an early state. Currently, the UK National Institute for Health and Care Excellence (NICE) and the American Society of Clinical Oncology (ASCO) recommend that surveillance consists of annual mammography and regular visits with physical examination and anamnesis [3, 4]. According to the Dutch guidelines, surveillance should consist of annual mammography and physical examination for at least 5 years after diagnosis [5].

Nevertheless, routine surveillance is not evidence based and the benefit of routine surveillance visits remains debatable. According to two systematic reviews, patients with asymptomatic- or mammography-detected LRRs or SPBCs have an improved survival compared to patients with symptomatic- or clinically detected LRRs or SPBCs [6, 7]. However, because of methodology constraints of the included studies, such as the absence of adjustment for confounders, additional studies are needed to better understand the effect of routine visits.

Routine visits have disadvantages. They (1) can lead to false positive results, (2) can increase levels of psychological distress and anxiety [8–10], (3) increase the burden on health care (costs) [11], and (4) detect a low percentage of the LRRs in the asymptomatic state. A Danish study showed that 2.0% of the initial mammograms and 1.6% of the subsequent mammograms in breast cancer survivors were false positive [12]. Those false positive results not only lead to unnecessary invasive biopsies and costs, but also to psychological distress. Previous studies performed in Austria and Slovenia showed that mammography and routine visits were causing distress in more than one third of the patients [13, 14]. Routine visits cost on average 253 euros ($258), and take around 30 min [11, 15, 16]. In the Netherlands approximately 77,000 women receive a routine visits each year [17], meaning approximately 19.5 million euros ($19.9 million) and 38,500 h are invested in routine visits annually. In case the follow-up would be based on individual risks of recurrence instead of on the Dutch guidelines—which would result in less routine follow-up visits—17.0–62.1% of the follow-up visits and 18.2–64.4% of the costs could be saved [11]. A Dutch study showed that, during the first 5 years of surveillance, only 34% of the LRRs were detected at routine visits in asymptomatic patients. Sixteen percent was detected at routine visits in symptomatic patients, and as much as 49% was detected at interval visits, i.e., in-between routine visits [18]. Only 2.6% of the patients develop a LRR within 5 years following breast cancer diagnosis, with individual risks depending on patient, tumor-, and treatment-related characteristics [19, 20]. With only 34% detected asymptomatically during routine visits, this means that 114 patients need to receive routine visits for 5 years, in order to detect one LRR at a routine visit in an asymptomatic patient.

As the benefit of routine surveillance visits is debatable and given the downsides of the visits, it is important to quantify the benefit and contribution of those visits to the early detection of LRRs and the prevention of distant metastases. Therefore, the current study aimed to compare the severity of the LRR (characterized by tumor size, tumor grade, and number of positive lymph nodes of the LRR) and the subsequent risk of a distant metastasis (DM) between LRRs detected at routine visits and interval visits.

## Methods

### Study population and data collection

Data were obtained from the Netherlands Cancer Registry (NCR). The NCR is a nationwide population-based registry, hosted by the Netherlands Comprehensive Cancer Organisation (IKNL), that includes data on all newly diagnosed cancer patients since 1989. Reports of newly diagnosed malignancies are obtained from the nationwide network and registry of histo- and cytopathology in the Netherlands (PALGA). Subsequently, trained data managers record information on patient, tumor, and treatment characteristics from the patients’ files. In addition, all first recurrences, both LRRs or DMs, within 5 years following diagnoses were registered for women diagnosed in 2004 or between 2006 and 2008. All recurrences within 10 years following diagnoses were registered for women diagnosed in 2003 or 2005. Vital status was obtained by linkage to the Municipal Personal Records database, which was complete until February 1st, 2021. This study was approved by the Privacy Review Board of the NCR (reference number K20.057).

**Fig. 1:**
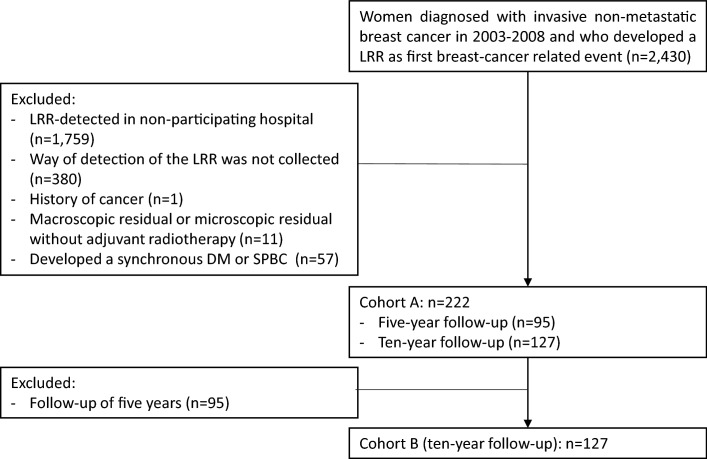
Flowchart of the study population. *DM* distant metastasis, *LRR* locoregional recurrence, *SPBC* second primary breast cancer

Patients were eligible if they were diagnosed with invasive non-metastatic breast cancer between 2003 and 2008, were surgically treated in a Dutch hospital, and developed a LRR as first breast cancer-related event after primary treatment (Fig. 1). Patients were ineligible if they developed a LRR with unknown way of detection, had been diagnosed with cancer (of any type, except basal cell and squamous cell skin cancer and carcinoma in situ of the cervix) before diagnosis of the primary breast tumor in 2003–2008, had macroscopic tumor left after surgery for their primary tumor or microscopic tumor without adjuvant radiotherapy, or were diagnosed with a DM or second primary tumor (of any type, except basal cell and squamous cell skin cancer and carcinoma in situ of the cervix) before or synchronous with (diagnosed within 30) diagnosis of the first LRR.

Additional data for this study were collected of a subset of LRR patients treated in one of the 15 participating hospitals. These 15 hospitals included both academic, teaching and general hospitals, providing a good representation of the breast cancer care in the Netherlands. The additionally collected data included way of detection of the LRR (routine or interval) and characteristics of the LRR (e.g., tumor size, tumor grade, positive nodes). Two cohorts were composed to answer our research questions. Cohort A was used to compare the severity of LRRs detected at routine visits and interval visits. This cohort consisted of all patients who met the in- and exclusion criteria and who were treated for their LRR in one of the 15 hospitals. Cohort B was used to investigate the association between way of detection of the LRR and risk of a subsequent DM. Patients from cohort A with a follow-up of 10 years were included in cohort B, as data on the development of recurrences after a LRR was previously collected for those patients.

### Definitions

LRRs include both local recurrences (LRs) and regional recurrences (RRs). A LR was defined as the reappearance of invasive cancer in the ipsilateral breast or chest wall [21]. A RR was defined as the reappearance of cancer in the ipsilateral axillary, infraclavicular, supraclavicular, internal mammary/parasternal, or intramammary lymph nodes [21]. Patients with a synchronous LR and RR (diagnosed within 30 days of each other) were registered as having both. A DM was defined as the recurrence of breast cancer in any other location except for the contralateral breast. A SPBC meant the patient was diagnosed with a new primary breast tumor, including those in the contralateral breast. Patients with a LRR detected at a planned surveillance mammography and/or clinical examination were defined as routine-LRR patients. Patients with a LRR detected at a self-requested visit or a visit not aimed at detecting recurrences, e.g., at the time of reconstructive breast surgery, were referred to as interval-LRR patients.

The development of a DM as first event after a LRR, was analyzed as the primary outcome. The development of a second LRR or a SPBC, or death, as first event after a LRR, were analyzed as secondary outcomes. If patients were diagnosed with multiple events within 30 days, the most severe event was registered as endpoint. DM was defined as most severe, followed by LRR and SPBC. Follow-up was calculated from the date of LRR diagnosis to date of event or the last date of observation.

The socioeconomic status (SES) of a patient was based on their postal code at the time of diagnosis. Estrogen receptor (ER) and progesterone receptor (PR) status were combined into the variable hormone receptor (HR) status (ER and/or PR positive, ER and PR negative). Tumor size and stage were based on pathological assessment for the primary tumor and on pathological or clinical assessment for the LRR, depending on whether the pathological assessment was available. The disease-free interval (DFI) was defined as the number of years between date of diagnosis of the primary tumor and date of diagnosis of the LRR.

### Statistical analysis

Patient-, primary tumor-, LRR-, and treatment-related characteristics of cohort A and B were summarized and patients were compared according to way of LRR-detection using Chi-squared and Wilcoxon rank-sum tests.

### Cohort A

Tumor size, tumor grade, and number of positive lymph nodes of the LRR were regarded as proxies for the severity of the LRR. As those characteristics differ between LRs and RRs it was decided to stratify the baseline characteristics by type of LRR.

### Cohort B

As some variables were missing, multiple imputation by chained equations (MICE) was used to impute missing values [22]. Missing values were related to coding rules which changed over time, and to missing data in the patient files. Missing values were assumed to be fully accounted for by measured (complete) variables and were therefore considered as missing at random. Variables used in the Cox regression analyses were used in the imputation process. Imputation was repeated 25 times, and the estimates and standard errors were pooled using Rubin’s rules [23]. For comparison, the analyses were performed with both the imputed datasets and the datasets only containing the complete cases.

Cause-specific cumulative incidence functions (CIFs) were calculated to estimate the probability of a DM, second LRR, SPBC, or death as first event after a LRR, specified by way of detection. CIFs calculate the crude risk of a certain event, while taking into account that a patient might have experienced one of the other events. Hence, they take the occurrence of competing events into account. Cox proportional hazard models were used to estimate cause-specific hazard ratios (cHRs) and 95% confidence intervals (CI) for the association between way of LRR-detection and risk of a subsequent DM, second LRR, SPBC, or death. cHRs take the occurrence of competing events into account. The development of a DM, second LRR, SPBC, or death was regarded as a competing event, when not regarded as the outcome of interest. Patients were censored at the occurrence of a competing event or at the last date of observation. The cHRs for the secondary outcomes, i.e., second LRR, SPBC, or death, were shown to give a complete overview of the association between way of LRR-detection and risk of a DM [24]. The selection of potential confounders was based on literature or on significant associations with the exposure and outcome in univariate analyses (*p* < 0.1). The following variables were selected as confounders: tumor size, tumor grade, lymph node status, and type of surgery of the primary tumor, and tumor grade, and lymph node status of the LRR. Log–log plots and scaled Schoenfeld residuals showed that all variables met the proportionality assumptions.

A sensitivity analysis was performed without patients with a DFI of 5 years or longer, as surveillance consist of annual mammography for at least 5 years after diagnosis in the Netherlands. A second sensitivity analysis was performed to investigate whether the risk of a subsequent DM differed between asymptomatic and symptomatic patients. This analysis was performed to investigate whether early diagnosis was beneficial for patients most likely to benefit from it, i.e., asymptomatic patients.

A two sided *p*-value < 0.05 was considered statistically significant. All data were analyzed with Stata version 16 software.

## Results

### Baseline characteristics

Cohort A included 109 routine-LRR patients (49.1%) and 113 interval-LRR patients (50.9%). The median DFI was comparable between interval-LRR (2.6 years, IQR 1.7–4.1) and routine-LRR patients (2.9 years, IQR 1.8–4.8). Interval-LRR patients had more often a grade 3 primary tumor (41.6% vs. 30.3%) and received less often radiotherapy as treatment for their primary tumor (45.1% vs. 59.6%) compared to routine-LRR patients (Table 1). Interval-LRR patients had more often a LRR detected by physical examination only (54.9% vs. 42.2%), were more often diagnosed with a RR (39.8% vs. 22.9%), and experienced more often symptoms (70.9% vs. 30.3%) compared to routine-LRR patients (Table 2). Cohort B included 66 routine-LRR patients (52.0%) and 61 interval-LRR patients (48.0%) (Table 1). The DFI was comparable between interval-LRR and routine-LRR patients [3.3 years (IQR 2.3–6.0) vs. 3.8 years (IQR 2.1–6.3)], as was the follow-up time from diagnosis of the LRR to a subsequent event (i.e., DM, second LRR, SPBC, death) or last observation [2.9 years, (IQR 0.9–5.8) vs. 2.8 years (IQR 1.2–5.4)].

**Table 1:** Patient-, tumor-, and treatment-related characteristics of the *primary tumor*, specified for cohort A and B, and by way of detection (*N* (%))

	Cohort A			Cohort B		
	*N* = 222			*N* = 127		
	LRR detected at routine visit	LRR detected at interval visit	*p* value^a^	LRR detected at routine visit	LRR detected at interval visit	*p* value^a^
Patients	109 (49.1)	113 (50.9)		66 (52.0)	61 (48.0)	
Age at diagnosis			0.87			0.74
< 40	7 (6.4)	9 (8.0)		4 (6.1)	5 (8.2)	
40–49	20 (18.4)	24 (21.2)		7 (10.6)	10 (16.4)	
50–75	69 (63.3)	69 (61.1)		46 (69.7)	38 (62.3)	
> 75	13 (11.9)	11 (9.7)		9 (13.6)	8 (13.1)	
SES			0.83			0.76
High	34 (31.2)	32 (28.3)		20 (30.3)	15 (24.6)	
Medium	44 (40.4)	45 (39.8)		24 (36.4)	23 (37.7)	
Low	31 (28.4)	36 (31.9)		22 (33.3)	23 (37.7)	
Number of mammograms per year			0.44			0.57
0	0 (0.00)	1 (0.9)		0 (0.0)	1 (1.6)	
1	51 (46.8)	56 (49.6)		38 (57.6)	30 (49.2)	
2	47 (43.1)	40 (35.4)		22 (33.3)	20 (32.8)	
3–4	6 (5.5)	3 (2.7)		3 (4.6)	1 (1.6)	
Unknown	5 (4.6)	13 (11.5)		3 (4.6)	9 (14.8)	
Histology			0.12			0.52
Ductal	83 (76.2)	97 (85.8)		50 (75.8)	51 (83.6)	
Lobular	21 (19.3)	11 (9.7)		12 (18.2)	9 (14.8)	
Mixed	1 (0.9)	3 (2.7)		1 (1.5)	0 (0.0)	
Other	4 (3.7)	2 (1.8)		3 (4.6)	1 (1.6)	
Tumor size			0.17			0.15
< 2 cm	62 (56.9)	55 (48.7)		38 (57.6)	25 (41.0)	
2–5 cm	44 (40.4)	51 (45.1)		25 (37.9)	33 (54.1)	
> 5 cm	2 (1.8)	7 (6.2)		2 (3.0)	3 (4.9)	
Unknown	1 (0.9)	0 (0.0)		1 (1.5)	0 (0.0)	
Tumor grade			0.03			0.10
1	26 (23.9)	13 (11.5)		14 (21.2)	5 (8.2)	
2	44 (40.4)	45 (39.8)		29 (43.9)	27 (44.3)	
3	33 (30.3)	47 (41.6)		19 (28.8)	24 (39.3)	
Unknown	6 (5.5)	8 (7.1)		4 (6.1)	5 (8.2)	
Positive nodes			0.42			0.01
0	71 (65.1)	66 (58.4)		46 (69.7)	34 (55.7)	
1–3	27 (24.8)	37 (32.7)		12 (18.2)	25 (41.0)	
> 3	11 (10.1)	10 (8.9)		8 (12.1)	2 (3.3)	
Stage			0.44			0.01
Stage I	47 (43.1)	40 (35.4)		32 (48.5)	18 (29.5)	
Stage II	48 (44.0)	59 (52.2)		24 (36.4)	38 (62.3)	
Stage III	14 (12.8)	14 (12.4)		10 (15.2)	5 (8.2)	
Multifocality			0.42			0.93
Yes	17 (15.6)	23 (20.4)		13 (19.7)	12 (19.7)	
No	81 (74.3)	82 (72.6)		50 (75.8)	48 (78.7)	
Unknown	11 (10.1)	8 (7.1)		3 (4.6)	1 (1.6)	
Hormonal status			0.45			0.85
ER + and/or PR +	80 (73.4)	79 (69.9)		50 (75.8)	47 (77.1)	
ER − and PR −	23 (21.1)	29 (25.7)		15 (22.7)	13 (21.3)	
Unknown	6 (5.5)	5 (4.4)		1 (1.5)	1 (1.6)	
HER2-Neu status			0.27			0.93
Positive	6 (5.5)	10 (8.9)		4 (6.1)	4 (6.6)	
Negative	56 (51.4)	51 (45.1)		30 (45.5)	28 (45.9)	
Unknown	47 (43.1)	52 (46.0)		32 (48.5)	29 (47.5)	
Type of surgery			0.11			0.04
Breast conserving surgery	56 (51.4)	46 (40.7)		35 (53.0)	21 (34.4)	
Mastectomy	53 (48.6)	67 (59.3)		31 (47.0)	40 (65.6)	
Chemotherapy			0.70			0.29
Yes	34 (31.2)	38 (33.6)		16 (24.2)	20 (32.8)	
No	75 (68.8)	75 (66.4)		50 (75.8)	41 (67.2)	
Endocrine therapy			0.12			0.05
Yes	28 (25.7)	40 (35.4)		17 (25.8)	26 (42.6)	
No	81 (74.3)	73 (64.6)		49 (74.2)	35 (57.4)	
Targeted therapy			0.95			0.61
Yes	5 (4.6)	5 (4.4)		2 (3.0)	1 (1.6)	
No	104 (95.4)	108 (95.6)		64 (97.0)	60 (98.4)	
Radiotherapy			0.03			0.01
Yes	65 (59.6)	51 (45.1)		41 (62.1)	23 (37.7)	
No	44 (40.4)	62 (54.9)		25 (37.9)	38 (62.3)	
Axillary lymph node dissection			0.22			0.22
Yes	51 (46.8)	62 (54.9)		34 (51.5)	38 (62.3)	
No	58 (53.2)	51 (45.1)		32 (48.5)	23 (37.7)	

*ER* estrogen receptor, *Her2neu* Human Epidermal growth factor Receptor 2, *LRR* locoregional recurrence, *PR* progesterone receptor, *SES* socioeconomic status. Percentages may not add up to 100% because of rounding

^a^Chi-squared test was used to compare patients with a LRR detected at a routine visit with patients with a LRR detected at an interval visit. The *p* value is calculated on known values only

**Table 2:** Patient-, tumor, and treatment-related characteristics of the *LRR*, specified for cohort A and B, and by way of detection (*N* (%))

	Cohort A			Cohort B		
	*N* = 222			*N* = 127		
	LRR detected at routine visit	LRR detected at interval visit	*p* value^a^	LRR detected at routine visit	LRR detected at interval visit	*p* value^a^
Patients	109 (49.1)	113 (50.9)		66 (52.0)	61 (48.0)	
Age at diagnosis of the LRR			0.69			1.00
< 40	4 (3.6)	6 (5.2)		3 (4.6)	3 (4.9)	
40–49	17 (15.5)	13 (11.3)		5 (7.6)	5 (8.2)	
50–75	70 (63.6)	79 (68.7)		43 (65.2)	40 (65.6)	
≥ 75	19 (17.3)	17 (14.8)		15 (22.7)	13 (21.3)	
Method of detection of the LRR			0.01			0.01
Physical examination	46 (42.2)	62 (54.9)		24 (36.4)	38 (62.3)	
Mammography	14 (12.8)	4 (3.5)		11 (16.7)	2 (3.3)	
Physical examination and mammography	18 (16.5)	11 (9.7)		12 (18.2)	7 (11.5)	
Unknown	31 (28.4)	36 (31.9)		19 (28.8)	14 (23.0)	
Disease-free interval (years) (Median, (IQR))	2.9 (1.8–4.8)	2.6 (1.7–4.1)	0.59	3.8 (2.1–6.3)	3.3 (2.3–6.0)	0.96
Type of recurrence			0.03			0.13
Local recurrence	79 (72.5)	64 (56.6)		46 (69.7)	39 (63.9)	
Regional recurrence	25 (22.9)	45 (39.8)		15 (22.7)	21 (34.4)	
Both	5 (4.6)	5 (3.5)		5 (7.6)	1 (1.6)	
Type of subsequent event						0.10
DM	NA	NA		16 (24.2)	17 (27.9)	
Second LRR	NA	NA		7 (10.6)	3 (4.9)	
SPBC	NA	NA		4 (6.1)	2 (3.3)	
Death	NA	NA		3 (4.6)	11 (18.0)	
Event free	NA	NA		36 (54.6)	28 (45.9)	
Symptoms related to the LRR			< 0.01			< 0.01
Yes	33 (30.3)	80 (70.9)		19 (28.8)	42 (68.9)	
No	61 (56.0)	18 (15.9)		37 (56.1)	9 (14.8)	
Unknown	15 (13.8)	15 (13.3)		10 (15.2)	10 (16.4)	
Histology of the LRR			0.24			0.25
Ductal	75 (68.8)	71 (62.8)		47 (71.2)	35 (57.4)	
Lobular	21 (19.3)	12 (10.6)		12 (18.2)	9 (14.8)	
Mixed	2 (1.8)	3 (2.7)		1 (1.5)	3 (4.9)	
Other	0 (0.0)	2 (1.8)		0 (0.0)	2 (3.3)	
Unknown	11 (10.1)	25 (22.1)		6 (9.1)	12 (19.7)	
Tumor size of the LRR			0.18			0.09
< 2 cm	71 (65.1)	79 (69.9)		43 (65.2)	42 (68.9)	
2–5 cm	29 (26.6)	21 (18.6)		20 (30.3)	12 (19.7)	
> 5 cm	1 (0.9)	4 (3.5)		0 (0.0)	3 (4.9)	
Unknown	8 (7.3)	9 (8.0)		3 (4.6)	4 (6.6)	
Tumor grade of the LRR			0.86			0.76
1	11 (10.1)	7 (6.2)		8 (12.1)	5 (8.2)	
2	33 (30.3)	22 (19.5)		16 (24.2)	14 (23.0)	
3	18 (16.5)	16 (13.3)		12 (18.2)	7 (11.5)	
Unknown	47 (43.1)	69 (61.1)		30 (45.5)	35 (57.4)	
Positive nodes of the LRR			0.10			0.01
0	62 (56.9)	56 (49.6)		37 (56.1)	37 (60.7)	
1–3	17 (15.6)	17 (15.0)		12 (18.2)	2 (3.3)	
> 3	10 (9.2)	22 (19.5)		4 (6.1)	11 (18.0)	
Unknown	20 (18.4)	18 (15.9)		13 (19.7)	11 (18.0)	
Multifocality of the LRR			0.12			0.86
Yes	7 (6.4)	14 (12.4)		6 (9.1)	6 (9.8)	
No	100 (91.7)	96 (85.0)		60 (90.9)	54 (88.5)	
Unknown	2 (1.8)	3 (2.7)		0 (0.0)	1 (1.6)	
Hormonal status of the LRR			0.69			0.86
ER + and/or PR +	68 (62.4)	67 (59.3)		43 (65.2)	40 (65.6)	
ER − and PR −	39 (35.8)	43 (38.1)		23 (34.9)	20 (32.8)	
Unknown	2 (1.8)	3 (2.7)		0 (0.0)	1 (1.6)	
HER2-Neu status of the LRR			0.18			1.00
Positive	15 (13.8)	23 (20.4)		11 (16.7)	10 (16.4)	
Negative	92 (84.4)	87 (77.0)		55 (83.3)	50 (82.0)	
Unknown	2 (1.8)	3 (2.7)		0 (0.0)	1 (1.6)	
Surgery of the LRR^b^			0.37			0.73
Yes	73 (67.0)	70 (62.0)		39 (59.1)	38 (62.3)	
No	14 (12.8)	19 (16.8)		11 (16.7)	9 (14.8)	
Unknown	22 (20.2)	24 (21.2)		16 (24.2)	14 (23.0)	
Chemotherapy of the LRR			0.64			0.68
Yes	18 (16.5)	21 (18.6)		10 (15.2)	11 (18.0)	
No	69 (63.3)	68 (60.2)		40 (60.6)	36 (59.0)	
Unknown	22 (20.2)	24 (21.2)		16 (24.2)	14 (23.0)	
Endocrine therapy of the LRR			0.77			0.78
Yes	41 (37.6)	40 (35.4)		22 (33.3)	22 (36.1)	
No	46 (42.2)	49 (43.4)		28 (42.4)	25 (41.0)	
Unknown	22 (20.2)	24 (21.2)		16 (24.2)	14 (23.0)	
Targeted therapy of the LRR			0.25			0.63
Yes	4 (3.7)	8 (7.1)		3 (4.6)	4 (6.6)	
No	83 (76.2)	81 (71.7)		47 (71.2)	43 (70.5)	
Unknown	22 (20.2)	24 (21.2)		16 (24.2)	14 (23.0)	
Radiotherapy of the LRR			0.02			0.03
Yes	36 (33.0)	53 (46.9)		21 (31.8)	30 (49.2)	
No	51 (46.8)	36 (31.9)		29 (43.9)	17 (27.9)	
Unknown	22 (20.2)	24 (21.2)		16 (24.2)	14 (23.0)	

*DM* distant metastasis, *ER* estrogen receptor, *Her2neu* Human Epidermal growth factor Receptor 2, *IQR* interquartile range, *LRR* locoregional recurrence, *PR* progesterone receptor, *SPBC* second primary breast cancer. Percentages may not add up to 100% because of rounding

^a^Chi-squared and Wilcoxon rank-sum test were used to compare patients with a LRR detected at a routine visit with patients with a LRR detected at an interval visit. The *p* value is calculated on known values only

^b^Surgery included both breast-conserving surgeries, mastectomies and axillary lymph node dissection

### Cohort A

Concerning the severity of the LRR, interval-LRs were more often smaller than 2 cm compared to routine-LRs (73.4% vs. 65.8%), although tumor size did not statistically differ between the two groups (*p* = 0.06) (Supplementary 1 and 2). Tumor grade did not differ between interval-detected LRs and routine-detected LRs (*p* = 0.84). Tumor grade and number of positive lymph nodes did not differ between interval-detected RRs and routine-detected RRs (*p* = 0.32 and *p* = 0.67, respectively).

### Cohort B

**Fig. 2:**
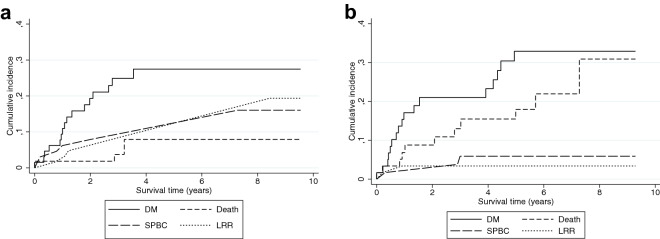
Cumulative incidence functions of the probability of developing a distant metastasis (DM), second locoregional recurrence (LRR), second primary breast cancer (SPBC), or death after a first LRR for patients with a LRR detected at a **a** routine visit or **b** interval visit (Cohort B)

Sixteen of the routine-LRR patients developed a DM (24.2%), 7 developed a second LRR (10.6%), 4 developed a SPBC (6.1%), 3 died (4.6%), and 36 were event free at the end of the 10 year follow-up period (54.6%). In comparison, 17 of the interval-LRR patients developed a DM (27.9%), 3 developed a second LRR (4.9%), 2 developed a SPBC (3.3%), 11 died (18.0%), and 28 were event free (45.9%). The CIF of developing a DM as first event within 5 years after diagnosis of a LRR was 27.5% (95% CI 16.6–39.5%) for routine-LRR patients and 32.9% (95% CI 20.4–46.0%) for interval-LRR patients (Fig. 2). Unadjusted competing-risk analysis showed no association between way of LRR-detection and risk of developing a DM (cHR: 1.19, 95% CI 0.60–2.36) (Table 3). After adjusting for confounders, the cHR remained insignificant (cHR: 1.22; 95% CI 0.49–3.06). Unadjusted competing-risk analysis showed that interval-LRR patients had a higher risk of death compared to routine-LRR patients (cHR: 3.84, 95% CI 1.07–13.79). The adjusted cHRs could not be calculated for the risk of a second LRR, SPBC, or death, because of the low number of events. Complete cases analysis resulted in the exclusion of 77 patients and in an adjusted cHR of 6.85 (95% CI 0.24–193.90). Limiting the analysis to patients with a DFI of less than 5 years showed no difference in the risk of a DM between routine- or interval-LRR patients (adjusted cHR: 1.53; 95% CI 0.52–4.52) (Supplementary Table 3). No difference in the risk of a DM was seen between asymptomatic and symptomatic patients (adjusted cHR: 1.46; 95% CI 0.51–4.20) (Supplementary Table 4). Summary statistics showed that the value distributions among the observed and imputed variables were comparable.

**Table 3:** Cause-specific hazard ratios and 95% confidence intervals for the association of way of detection of the LRR (routine or interval surveillance visit) with risk of development of a distant metastasis (Cohort B)

	Distant metastasis		Second locoregional recurrence^a^	Second primary breast cancer^a^	Death^a^
	Crude	Adjusted	Crude	Crude	Crude
	HR (95% CI)	HR (95% CI)	HR (95% CI)	HR (95% CI)	HR (95% CI)
Way of detection					
LRR detected at routine visit	1.00 (reference)	1.00 (reference)	1.00 (reference)	1.00 (reference)	1.00 (reference)
LRR detected at interval visit	1.19 (0.60–2.36)	1.22 (0.49–3.06)	0.47 (0.12–1.82)	0.57 (0.11–3.14)	3.84 (1.07–13.79)
Tumor size					
< 2 cm	1.00 (reference)	1.00 (reference)	1.00 (reference)	1.00 (reference)	1.00 (reference)
2–5 cm	2.57 (1.20–5.48)	1.65 (0.68–4.02)	1.63 (0.43–6.13)	1.58 (0.26–9.74)	1.32 (0.45–3.82)
> 5 cm	6.18 (1.33–28.74)	5.22 (0.78–35.05)	7.24 (0.78–66.97)	–	–
Tumor grade					
1	1.00 (reference)	1.00 (reference)	1.00 (reference)	1.00 (reference)	1.00 (reference)
2	1.26 (0.35–4.57)	1.13 (0.22–5.73)	–	0.98 (0.10–9.48)	0.76 (0.19–3.09)
3	3.55 (1.02–12.31)	2.17 (0.33–14.43)	–	0.98 (0.09–10.96)	1.03 (0.24–4.42)
Positive nodes					
0	1.00 (reference)	1.00 (reference)	1.00 (reference)	1.00 (reference)	1.00 (reference)
1–3	1.25 (0.59–2.68)	0.69 (0.27–1.78)	2.42 (0.60–9.68)	0.62 (0.06–6.14)	1.59 (0.52–4.91)
> 3	2.12 (0.62–7.23)	1.42 (0.34–6.01)	8.25 (1.42–47.88)	9.70 (1.50–62.66)	2.69 (0.33–22.11)
Type of surgery					
Breast conserving surgery	1.00 (reference)	1.00 (reference)	1.00 (reference)	1.00 (reference)	1.00 (reference)
Mastectomy	3.53 (1.53–8.14)	2.79 (1.07–7.25)	2.17 (0.56–8.42)	0.34 (0.06–2.00)	1.70 (0.56–5.17)
Grade of the LRR					
1	1.00 (reference)	1.00 (reference)	1.00 (reference)	1.00 (reference)	1.00 (reference)
2	1.66 (0.29–9.50)	1.30 (0.16–10.46)	–	–	0.65 (0.14–3.13)
3	2.29 (0.42–12.55)	1.36 (0.13–14.69)	–	–	0.41 (0.04–3.98)
Positive nodes of the LRR					
0	1.00 (reference)	1.00 (reference)	1.00 (reference)	1.00 (reference)	1.00 (reference)
1–3	3.01 (1.19–7.66)	2.99 (0.99–9.02)	5.37 (1.01–28.44)	6.73 (0.85–53.26)	1.01 (0.13–7.80)
> 3	2.33 (0.89–6.06)	1.54 (0.46–5.16)	5.10 (0.95–27.39)	3.40 (0.33–35.54)	–

The development of a DM, second LRR, SPBC, or death was regarded as a competing event, when not regarded as the outcome of interest. Patients were censored at the occurrence of a competing event or at the last date of observation. The cHRs for the secondary outcomes, i.e., second LRR, SPBC, or death, were shown to give a complete overview of the association between way of LRR-detection and risk of a DM

*CI* Confidence interval, *cHR* Cause-specific hazard ratio, *DM* Distant metastasis, *LRR* Locoregional recurrence, *SPBC* Second primary breast cancer

^a^Too little events occurred to calculate the adjusted cHR

## Discussion

In our study, no significant association was found between way of detection of the LRR and the severity of the LRR or the risk of a subsequent DM. A possible explanation for the absence of an association between way of LRR-detection and risk of a DM could be the increased awareness of patients with a history of breast cancer. This would increase the chance of a patient discovering the LRR themselves, and hence would lead to a weaker or no association between way of LRR-detection and risk of a DM. This hypothesis is supported by a study showing that 49% of the LRRs are detected at interval visits [18] and by the finding of the current study that interval-detected LRs tended to be smaller compared to routine-detected LRs, although this was not significant. The smaller size of interval-LRs might suggest that a group of patients are indeed very careful and check their breast more often, leading to earlier detection.

The lack of an association might also be explained by studies showing that mainly the more aggressive and drug resistant tumors recur [25–27]. A study performed by our group, showed that 49% of the patients with a LRR were still alive 10 years after development of the primary tumor, compared to 84% of the patients without a LRR [28]. This suggests that primary tumors leading to LRRs might be more aggressive from the onset, which could explain the lack of a beneficial effect of routine visits. Seventy-two percent of the patients with a SPBC were still alive 10 years after development of the primary tumor [28], suggesting that SPBCs might be less aggressive than the primary tumors leading to LRRs. As the way of detection of the SPBC was not known in the current study, we were not able to investigate the effect of early detection of SPBC.

Two systematic reviews showed that patients with asymptomatic- or mammography-detected LRRs or SPBCs have an improved survival compared to patients with symptomatic- or clinically detected LRRs or SPBCs [6, 7]. Results could differ from our study as most of the studies included in the reviews included patients diagnosed before 2003. As treatments and diagnostics have been drastically improved over time, results are likely to be different in a more contemporary population. Trastuzumab has for instance been introduced in 2005, and it has been shown that this decreased the risk of LRRs [29–31]. A previous study, showing a decreased risk of LRRs over the years, suggested that the developments in systemic treatment might in a large extent be responsible for the decreased risk of LRRs [32]. Furthermore, in contrast to the more exploratory studies that were described in the systematic reviews, our study included a multivariable analysis in which we additionally controlled for patient-, tumor-, and treatment-related characteristics.

The unadjusted competing-risk analysis showed that interval-LRR patients had a higher risk of death compared to routine-LRR patients, resulting in less interval-LRR patients being at risk of developing a DM. This lower number of interval-LRR patients could also be an explanation for the lack of an association. The competing-risk analyses showing the risk of developing one of the secondary outcomes (i.e., a second LRR, SPBC, or death), cannot be used to draw a conclusion about the protective effect of routine visits on the development of a secondary outcome, since these analyses were unadjusted. In addition, no conclusion can be drawn about the protective effect of routine visits on the risk of death, since patients were censored if they developed a DM, second LRR, or SPBC. The analyses showing the risk of developing one of the secondary outcomes were only performed to provide a complete overview of the risk of developing a DM.

Interval-LRR patients received more often endocrine therapy for their primary tumor compared to routine-LRR patients. Patients receiving endocrine therapy regularly visit the medical oncologist. These visits are not regarded as routine surveillance visits, as they are rarely aimed at detecting recurrences. However, it is possible that patients experiencing symptoms might ask their medical oncologist for a physical examination during these visits or that the medical oncologist performs a physical examination as routine. Furthermore, routine-LRR patients received more often radiotherapy as treatment for their primary tumor compared to interval-LRR patients. A previous Dutch study showed that patients receiving radiotherapy received more routine surveillance visits compared to patients not receiving radiotherapy [33]. Hence, the LRRs of patients receiving radiotherapy might more often be diagnosed at routine visits. Future research should register when and by which specialism the LRR had been diagnosed for better interpretation of the results.

In our study 24.2% of the routine- and 27.9% of the interval-LRR patients developed a DM within a median of 2.8 and 2.9 years of follow-up, respectively. Previous studies showed that approximately 21% of the lymph node negative patients with a LRR and 38% of the lymph node positive patients with a LRR developed a subsequent DM within 3 years of follow-up [34, 35]. In our study 70.0% of the patients were lymph node negative and 30.0% were lymph node positive. When using the numbers of the studies above it would be expected that approximately (0.21 × 70 + 0.38 + 30 =) 26.1% of the patients will develop a DM within 3 years of follow-up, which is according to our results.

The main sources of bias in studies investigating the beneficial effect of early detection are lead and length time bias. Lead time bias means that patients with a routine-LRR live longer than patients with a self-detected interval-LRR, even when earlier detection did not change the natural course of the disease. Length time bias means that the less aggressive, slowly growing, tumors are more likely to be detected at routine visits and the more aggressive, faster growing, tumors at interval visits. However, since the median DFI, the IQR of the DFI, and the tumor characteristics hardly differed between the group of patients with a routine- or interval-LRR, we believe that it is unlikely that lead or length time bias was present.

Strengths of this study include the availability of data on a large number of variables, the relatively long follow-up and the use of cause-specific hazard ratios to take the occurrence of competing events into account [24]. A limitation of the current study includes the small sample size and low number of events, which may have resulted in limited power. Furthermore, it is often stated that there should be at least ten events per variable included in the multivariable analyses. Although, in our study there were 4.9 events per variable, we think that it is unlikely that this low number of events will cause severe problems with the results [36], especially since the confidence intervals of our main analyses are not too wide. The missing values of part of the population can also be seen as a limitation of the current study. Multiple imputation was used to impute missing values, to avoid biased results and loss of power [37]. Furthermore, some of the interval-LRRs were not detected due to symptoms, and these LRRs might have been less aggressive than the average interval-LRR. However, as the sensitivity analysis showed no difference in the risk of a DM between symptomatic and asymptomatic patients, a possible decreased aggressiveness of some of the interval-LRRs is not expected to have influenced the results. Latest treatment developments were not reflected in the current study because of the inclusion of patients diagnosed over 15 years ago, which could have led to an overestimation of the effect of routine visits.

To conclude, our study showed that routine surveillance visits did not lead to less severe LRRs and did not decrease the risk of a subsequent DM. We therefore advise clinicians to plan surveillance visits in close consultation with the patient’s wishes and their risk on a LRR.

## Supplementary Information

Below is the link to the electronic supplementary material.

Supplementary file1 (docx 62.9 KB)

## Data Availability

The datasets generated during and analyzed during the current study will be made available via the NCR upon request and after approval of a proposal from the date of publication. The plan for the statistical analysis will be made available by the corresponding author upon request.
