# Supplementary material for: Routine and interval detection of locoregional breast cancer recurrences and risk of subsequent distant metastasis
Source: Breast Cancer Res Treat. 2022 Oct 31;197(1):123–35. doi: 10.1007/s10549-022-06757-3 (PMC9823019; doi:10.1007/s10549-022-06757-3)
Supplement: Supplementary file 1 — Supplementary file1 (docx 62.9 KB) [file 10549_2022_6757_MOESM1_ESM.docx]

Supplementary Information

Routine and interval detection of locoregional breast cancer recurrences and risk of subsequent distant metastasis

Breast Cancer Research and Treatment

Anouk H. Eijkelboom^1,2^, Linda de Munck^1^, Maaike de Vries^2^, , Anne Brecht Francken^3^, Mathijs P. Hendriks^4^, Luc Strobbe^5^, Annemieke Witteveen^6^, Marissa van Maaren^1,2^, Sabine Siesling^1,2^

1. Department of Research and Development, Netherlands Comprehensive Cancer Organisation (IKNL), Utrecht, the Netherlands
2. Department of Health Technology and Services Research, Technical Medical Centre, University of Twente, Enschede, the Netherlands
3. Department of Surgical Oncology, Isala Clinics, Zwolle, the Netherlands
4. Department of Medical Oncology, Northwest Clinics, Alkmaar, the Netherlands
5. Department of Surgical Oncology, Canisius Wilhelmina Hospital, Nijmegen, the Netherlands
6. Department of Biomedical Signals and Systems, Technical Medical Centre, University of Twente, Enschede, the Netherlands

**Corresponding author:**

Anouk H. Eijkelboom

[a.h.eijkelboom@utwente.nl](mailto:a.h.eijkelboom@utwente.nl)

Supplementary Tables

Supplementary Table 1 Patient-, tumor, and treatment-related characteristics of the *primary tumor* of patients in cohort A, specified by way of detection and type of recurrence (N (%))^a^.

|  | **LR detected at routine visit** | **LR detected at interval visit** | **P-value^b^** | **RR detected at routine visit** | **RR detected at interval visit** | **P-value^b^** |
| --- | --- | --- | --- | --- | --- | --- |
| **Patients** | 79 (55.2) | 64 (44.8) |  | 25 (35.7) | 45 (64.3) |  |
| **Age at diagnosis** |  |  | 0.87 |  |  | 0.86 |
| <40 | 4 (5.1) | 2 (3.1) |  | 3 (12.0) | 7 (15.6) |  |
| 40–49 | 16 (20.3) | 16 (25.0) |  | 3 (12.0) | 8 (17.8) |  |
| 50–75 | 50 (63.3) | 39 (60.9) |  | 17 (68.0) | 26 (57.8) |  |
| >75 | 9 (11.4) | 7 (10.9) |  | 2 (8.0) | 4 (8.9) |  |
| **SES** |  |  | 0.64 |  |  | 0.59 |
| High | 28 (35.4) | 18 (28.1) |  | 5 (20.0) | 13 (28.9) |  |
| Medium | 28 (35.4) | 26 (40.6) |  | 13 (52.0) | 18 (40.0) |  |
| Low | 23 (29.1) | 20 (31.3) |  | 7 (28.0) | 14 (31.1) |  |
| **Number of mammograms per year** |  |  | 0.48 |  |  | 0.21 |
| 0 | 0 (0.0) | 1 (1.6) |  | 0 (0.0) | 0 (0.0) |  |
| 1 | 40 (50.6) | 31 (48.4) |  | 8 (32.0) | 23 (51.1) |  |
| 2 | 31 (39.2) | 22 (34.4) |  | 15 (60.0) | 17 (37.8) |  |
| 4 | 4 (5.1) | 1 (1.6) |  | 1 (4.0) | 1 (2.2) |  |
| Unknown | 4 (5.1) | 9 (14.1) |  | 1 (4.0) | 4 (8.9) |  |
| **Histology** |  |  | 0.30 |  |  | 0.09 |
| Ductal | 59 (74.7) | 52 (81.3) |  | 19 (76.0) | 42 (93.3) |  |
| Lobular | 16 (20.3) | 7 (10.9) |  | 5 (20.0) | 3 (6.7) |  |
| Mixed | 1 (1.3) | 3 (4.7) |  | 0 (0.0) | 0 (0.0) |  |
| Other | 3 (3.8) | 2 (3.1) |  | 1 (4.0) | 0 (0.0) |  |
| **Tumor size** |  |  | 0.13 |  |  | 1.00 |
| <2 cm | 48 (60.8) | 31 (48.4) |  | 13 (52.0) | 23 (51.1) |  |
| 2–5 cm | 29 (36.7) | 29 (45.3) |  | 11 (44.0) | 20 (44.4) |  |
| >5 cm | 1 (1.3) | 4 (6.3) |  | 1 (4.0) | 2 (4.4) |  |
| Unknown | 1 (1.3) | 0 (0.0) |  | 0 (0.0) | 0 (0.0) |  |
| **Tumor grade** |  |  | 0.28 |  |  | 0.12 |
| 1 | 20 (25.3) | 10 (15.6) |  | 6 (24.0) | 3 (6.7) |  |
| 2 | 34 (43.0) | 29 (45.3) |  | 9 (36.0) | 14 (31.1) |  |
| 3 | 19 (24.1) | 21 (32.8) |  | 10 (40.0) | 24 (53.3) |  |
| Unknown | 6 (7.6) | 4 (6.3) |  | 0 (0.0) | 4 (8.9) |  |
| **Positive nodes** |  |  | 0.61 |  |  | 0.40 |
| 0 | 50 (63.3) | 37 (57.8) |  | 19 (76.0) | 27 (60.0) |  |
| 1–3 | 20 (25.3) | 21 (32.8) |  | 5 (20.0) | 15 (33.3) |  |
| >3 | 9 (11.4) | 6 (9.4) |  | 1 (4.0) | 3 (6.7) |  |
| **Stage** |  |  | 0.37 |  |  | 0.81 |
| Stage I | 34 (43.0) | 21 (32.8) |  | 12 (48.0) | 18 (40.0) |  |
| Stage II | 34 (43.0) | 35 (54.7) |  | 11 (44.0) | 23 (51.1) |  |
| Stage III | 11 (13.9) | 8 (12.5) |  | 2 (8.0) | 4 (8.9) |  |
| **Multifocality** |  |  | 0.16 |  |  | 0.50 |
| Yes | 14 (17.7) | 18 (28.1) |  | 1 (4.0) | 4 (8.9) |  |
| No | 57 (72.2) | 41 (64.1) |  | 21 (84.0) | 39 (86.7) |  |
| Unknown | 8 (10.1) | 5 (7.8) |  | 3 (12.0) | 2 (4.4) |  |
| **Hormonal status** |  |  | 0.77 |  |  | 0.77 |
| ER+ and/or PR+ | 60 (76.0) | 49 (76.6) |  | 17 (68.0) | 29 (64.4) |  |
| ER- & PR- | 14 (17.7) | 13 (20.3) |  | 7 (28.0) | 14 (31.1) |  |
| Unknown | 5 (6.3) | 2 (3.1) |  | 1 (4.0) | 2 (4.4) |  |
| **HER2-Neu status** |  |  | 0.66 |  |  | 0.05 |
| Positive | 4 (5.1) | 2 (3.1) |  | 0 (0.0) | 7 (15.6) |  |
| Negative | 43 (54.4) | 32 (50.0) |  | 11 (44.0) | 17 (37.8) |  |
| Unknown | 32 (40.5) | 30 (46.9) |  | 14 (56.0) | 21 (46.7) |  |
| **Type of surgery** |  |  | 0.05 |  |  | 0.61 |
| Breast-conserving surgery | 45 (57.0) | 26 (40.6) |  | 9 (36.0) | 19 (42.2) |  |
| Mastectomy | 34 (43.0) | 38 (59.4) |  | 16 (64.0) | 26 (57.8) |  |
| **Chemotherapy** |  |  | 0.26 |  |  | 0.03 |
| Yes | 28 (35.4) | 17 (26.6) |  | 4 (16.0) | 19 (42.2) |  |
| No | 51 (64.6) | 47 (73.4) |  | 21 (84.0) | 26 (57.8) |  |
| **Endocrine therapy** |  |  | 0.42 |  |  | 0.09 |
| Yes | 21 (26.6) | 21 (32.8) |  | 5 (20.0) | 18 (40.0) |  |
| No | 58 (73.4) | 43 (67.2) |  | 20 (80.0) | 27 (60.0) |  |
| **Targeted therapy** |  |  | 0.26 |  |  | 0.19 |
| Yes | 4 (5.1) | 1 (1.6) |  | 0 (0.0) | 3 (6.7) |  |
| No | 75 (94.9) | 63 (98.4) |  | 25 (100.0) | 42 (93.3) |  |
| **Radiotherapy** |  |  | 0.01 |  |  | 0.70 |
| Yes | 51 (64.6) | 27 (42.2) |  | 11 (44.0) | 22 (48.9) |  |
| No | 28 (35.4) | 37 (57.8) |  | 14 (56.0) | 23 (51.1) |  |
| **Axillary lymph node dissection** |  |  | 0.39 |  |  | 0.06 |
| Yes | 40 (49.4) | 37 (57.8) |  | 7 (28.0) | 23 (51.1) |  |
| No | 39 (50.6) | 27 (42.2) |  | 18 (72.0) | 22 (48.9) |  |
| ER: estrogen receptor, Her2neu: Human Epidermal growth factor Receptor 2, LRR: locoregional recurrence, PR: progesterone receptor, SES: socioeconomic status. Percentages may not add up to 100% because of rounding.   1. Numbers do not add up to 222, as patients with both a LR and RR are not included in the table. 2. Chi-squared test was used to compare patients with a LR/RR detected at a routine visit with patients with a LR/RR detected at an interval visit. The p-value is calculated on known values only. | | | | | | |

Supplementary Table 2 Patient-, tumor, and treatment-related characteristics of the *LRR* of patients in cohort A, specified by way of detection and type of recurrence (N (%))^a^.

|  | **LR detected at routine visit** | **LR detected at interval visit** | **P-value^b^** | **RR detected at routine visit** | **RR detected at interval visit** | **P-value^b^** |
| --- | --- | --- | --- | --- | --- | --- |
| **Patients** | 79 (55.2) | 64 (44.8) |  | 25 (35.7) | 45 (64.3) |  |
| **Age at diagnosis of the LRR** |  |  | 0.66 |  |  | 0.95 |
| <40 | 2 (2.5) | 2 (3.1) |  | 2 (8.0) | 4 (8.9) |  |
| 40–49 | 13 (16.5) | 6 (9.4) |  | 3 (12.0) | 6 (13.3) |  |
| 50–75 | 51 (64.6) | 44 (68.8) |  | 16 (64.0) | 30 (66.7) |  |
| ≥75 | 13 (16.5) | 12 (18.8) |  | 4 (16.0) | 5 (11.1) |  |
| **Method of detection of the LRR** |  |  | <0.01 |  |  | 0.13 |
| Physical examination | 24 (30.4) | 35 (54.7) |  | 21 (84.0) | 26 (57.8) |  |
| Mammography | 14 (17.7) | 3 (4.7) |  | 0 (0.0) | 3 (6.7) |  |
| Physical examination and mammography | 17 (21.5) | 8 (12.5) |  | 4 (16.0) | 16 (35.6) |  |
| Unknown | 24 (30.4) | 18 (28.1) |  |  |  |  |
| **Disease-free interval (years) (Median, (IQR))** | 3.05 (2.11 – 5.08) | 2.57 (1.78 – 4.04) | 0.10 | 2.17 (1.07 – 3.51) | 2.89 (1.66 – 4.11) | 0.09 |
| **Symptoms related to the LRR** |  |  | <0.01 |  |  | 0.04 |
| Yes | 17 (21.5) | 46 (71.9) |  | 14 (56.0) | 34 (75.6) |  |
| No | 51 (64.6) | 9 (14.1) |  | 9 (36.0) | 5 (11.1) |  |
| Unknown | 11 (13.9) | 9 (14.1) |  | 2 (8.0) | 6 (13.3) |  |
| **Histology of the LRR** |  |  | 0.42 |  |  | 0.52 |
| Ductal | 57 (72.2) | 47 (73.4) |  | 14 (56.0) | 23 (51.1) |  |
| Lobular | 17 (21.5) | 8 (12.5) |  | 4 (16.0) | 3 (6.7) |  |
| Mixed | 2 (2.5) | 2 (3.1) |  | 0 (0.0) | 1 (2.2) |  |
| Other | 0 (0.0) | 1 (1.6) |  | 0 (0.0) | 1 (2.2) |  |
| Unknown | 3 (3.8) | 6 (9.4) |  | 7 (28.0) | 17 (37.8) |  |
| **Tumor size of the LRR** |  |  | 0.06 |  |  | 0.82 |
| <2 cm | 52 (65.8) | 47 (73.4) |  | 16 (64.0) | 31 (68.9) |  |
| 2–5 cm | 23 (29.1) | 10 (15.6) |  | 4 (16.0) | 10 (22.2) |  |
| >5 cm | 0 (0.0) | 2 (3.1) |  | 1 (4.0) | 1 (2.2) |  |
| Unknown | 4 (5.1) | 5 (7.8) |  | 4 (16.0) | 3 (6.7) |  |
| **Tumor grade of the LRR** |  |  | 0.84 |  |  | 0.32 |
| 1 | 9 (11.4) | 6 (9.4) |  | 2 (8.0) | 1 (2.2) |  |
| 2 | 28 (35.4) | 19 (29.7) |  | 4 (16.0) | 3 (6.7) |  |
| 3 | 16 (20.3) | 8 (12.5) |  | 2 (8.0) | 6 (13.3) |  |
| Unknown | 26 (32.9) | 31 (48.4) |  | 17 (68.0) | 35 (77.8) |  |
| **Positive nodes of the LRR** |  |  |  |  |  | 0.67 |
| 0 | n/a | n/a |  | 5 (20.0) | 8 (17.8) |  |
| 1 - 3 |  |  |  | 9 (36.0) | 12 (26.7) |  |
| >3 |  |  |  | 8 (32.0) | 18 (40.0) |  |
| Unknown |  |  |  | 3 (12.0) | 7 (15.6) |  |
| **Multifocality of the LRR** |  |  | 0.31 |  |  | 0.23 |
| Yes | 5 (6.3) | 7 (10.9) |  | 1 (4.0) | 6 (13.3) |  |
| No | 73 (92.4) | 55 (85.9) |  | 23 (92.0) | 39 (86.7) |  |
| Unknown | 1 (1.3) | 2 (3.1) |  | 1 (4.0) | 0 (0.0) |  |
| **Hormonal status of the LRR** |  |  | 0.82 |  |  | 0.68 |
| ER+ and/ or PR+ | 54 (68.4) | 44 (28.1) |  | 11 (44.0) | 23 (51.1) |  |
| ER- & PR- | 24 (30.4) | 18 (28.1) |  | 13 (52.0) | 22 (48.9) |  |
| Unknown | 1 (1.3) | 2 (3.1) |  | 1 (4.0) | 0 (0.0) |  |
| **HER2-Neu status of the LRR** |  |  | 0.99 |  |  | 0.03 |
| Positive | 10 (12.7) | 8 (12.5) |  | 2 (8.0) | 14 (31.1) |  |
| Negative | 68 (86.1) | 54 (84.4) |  | 22 (88.0) | 31 (68.9) |  |
| Unknown | 1 (1.3) | 2 (3.1) |  | 1 (4.0) | 0 (0.0) |  |
| **Surgery of the LRR^c^** |  |  | 0.79 |  |  | 0.27 |
| Yes | 54 (68.4) | 46 (71.9) |  | 18 (72.0) | 22 (48.9) |  |
| No | 6 (7.6) | 6 (9.4) |  | 5 (20.0) | 12 (26.7) |  |
| Unknown | 19 (24.1) | 12 (18.8) |  | 2 (8.0) | 11 (24.4) |  |
| **Chemotherapy of the LRR** |  |  | 0.23 |  |  | 0.21 |
| Yes | 9 (11.4) | 4 (6.3) |  | 7 (28.0) | 16 (35.6) |  |
| No | 51 (64.6) | 48 (75.0) |  | 16 (64.0) | 18 (40.0) |  |
| Unknown | 19 (24.1) | 12 (18.8) |  | 2 (8.0) | 11 (24.4) |  |
| **Endocrine therapy of the LRR** |  |  | 0.62 |  |  | 0.08 |
| Yes | 26 (32.9) | 25 (39.1) |  | 15 (60.0) | 14 (31.1) |  |
| No | 34 (43.0) | 27 (42.2) |  | 8 (32.0) | 20 (44.4) |  |
| Unknown | 19 (24.1) | 12 (18.8) |  | 2 (8.0) | 11 (24.4) |  |
| **Targeted therapy of the LRR** |  |  | 0.35 |  |  | 0.08 |
| Yes | 1 (1.3) | 0 (0.0) |  | 1 (4.0) | 7 (15.6) |  |
| No | 59 (74.7) | 52 (81.3) |  | 22 (88.0) | 27 (60.0) |  |
| Unknown | 19 (24.1) | 12 (18.8) |  | 2 (8.0) | 11 (24.4) |  |
| **Radiotherapy of the LRR** |  |  | 0.07 |  |  | 0.24 |
| Yes | 22 (27.9) | 28 (43.8) |  | 12 (48.0) | 23 (51.1) |  |
| No | 38 (48.1) | 24 (37.5) |  | 11 (44.0) | 11 (24.4) |  |
| Unknown | 19 (24.1) | 12 (18.8) |  | 2 (8.0) | 11 (24.4) |  |
| ER: estrogen receptor, Her2neu: Human Epidermal growth factor Receptor 2, IQR: interquartile range, LRR: locoregional recurrence, n/a: not applicable, PR: progesterone receptor. Percentages may not add up to 100% because of rounding.   1. Numbers do not add up to 222, as patients with both a LR and RR are not included in the table. 2. Chi-squared and Wilcoxon rank sum test were used to compare patients with a LR/RR detected at a routine visit with patients with a LR/RR detected at an interval visit. The p-value is calculated on known values only. 3. Surgery included both breast-conserving surgeries, mastectomies and axillary lymph node dissection | | | | | | |

Supplementary Table 3 Cause-specific hazard ratios and 95% confidence intervals for the association of way of detection of the LRR (routine or interval surveillance visit) with risk of development of a distant metastasis in patients with a DFI of less than 5 years (n=82)^a^

|  | Distant metastasis | | Second locoregional recurrence^b^ | Second primary breast cancer^b^ | Death^b^ |
| --- | --- | --- | --- | --- | --- |
|  | Crude  HR (95% CI) | Adjusted  HR (95% CI) | Crude  HR (95% CI) | Crude  HR (95% CI) | Crude  HR (95% CI) |
| Way of detection |  |  |  |  |  |
| LRR detected at routine visit | 1.00 (reference) | 1.00 (reference) | 1.00 (reference) | 1.00 (reference) | 1.00 (reference) |
| LRR detected at interval visit | 1.08 (0.52 – 2.25) | 1.53 (0.52 – 4.52) | 0.39 (0.08 – 2.04) | 0.53 (0.10 – 2.88) | 7.28 (0.91 – 58.33) |
| Tumor size |  |  |  |  |  |
| <2 cm | 1.00 (reference) | 1.00 (reference) | 1.00 (reference) | 1.00 (reference) | 1.00 (reference) |
| 2–5 cm | 2.63 (1.14 – 6.05) | 1.83 (0.70 – 4.81) | 0.84 (0.18 – 3.80) | 1.41 (0.23 – 8.67) | 0.95 (0.25 – 3.64) |
| >5 cm | 27.50 (5.24 – 144.35) | 77.44 (5.04 – 1188.88) | - | - | - |
| Tumor grade |  |  |  |  |  |
| 1 | 1.00 (reference) | 1.00 (reference) | 1.00 (reference) | 1.00 (reference) | 1.00 (reference) |
| 2 | 1.18 (0.31– 4.51) | 1.70 (0.21 – 13.73) | - | 1.27 (0.13 – 12.31) | 0.45 (0.09 – 2.24) |
| 3 | 3.00 (0.86 – 10.43) | 2.59 (0.22 – 30.53) | - | 0.88 (0.08 – 9.83) | 0.56 (0.11 – 2.85) |
| Positive nodes |  |  |  |  |  |
| 0 | 1.00 (reference) | 1.00 (reference) | 1.00 (reference) | 1.00 (reference) | 1.00 (reference) |
| 1–3 | 1.69 (0.75 – 3.78) | 0.89 (0.33 – 2.35) | 1.46 (0.27 – 8.00) | 0.78 (0.08 – 8.11) | 0.78 (0.16 – 3.82) |
| >3 | 2.46 (0.71 – 8.54) | 2.61 (0.58 – 11.82) | 3.60 (0.38 – 34.09) | 10.36 (1.58 – 68.12) | - |
| Type of surgery |  |  |  |  |  |
| Breast conserving | 1.00 (reference) | 1.00 (reference) | 1.00 (reference) | 1.00 (reference) | 1.00 (reference) |
| Mastectomy | 3.11 (1.26 – 7.65) | 2.96 (0.98 – 8.96) | 1.92 (0.37 – 9.95) | 0.30 (0.05 – 1.72) | 2.93 (0.59 – 14.46) |
| Grade of the LRR |  |  |  |  |  |
| 1 | 1.00 (reference) | 1.00 (reference) | 1.00 (reference) | 1.00 (reference) | 1.00 (reference) |
| 2 | 1.90 (0.29 – 12.43) | 1.50 (0.15 – 14.91) | - | - | 0.51 (0.07 – 3.55) |
| 3 | 2.78 (0.48 – 16.22) | 1.98 (0.13 – 30.59) | - | - | 0.36 (0.04 – 3.32) |
| Positive nodes of the LRR |  |  |  |  |  |
| 0 | 1.00 (reference) | 1.00 (reference) | 1.00 (reference) | 1.00 (reference) | 1.00 (reference) |
| 1–3 | 2.61 (0.95 – 7.15) | 3.40 (0.88 – 13.21) | 6.75 (0.93 – 49.00) | 6.24 (0.78 – 49.67) | 1.10 (0.13 – 9.48) |
| >3 | 1.95 (0.70 – 5.40) | 0.96 (0.26 – 3.53) | 5.84 (0.83 – 41.09) | 2.84 (0.27 – 29.86) | - |
| CI: confidence interval, cHR: cause-specific hazard ratio, DM: distant metastasis, DFI: disease-free interval, LRR: locoregional recurrence, SPBC: second primary breast cancer.   1. The development of a DM, second LRR, SPBC, or death was regarded as a competing event, when not regarded as the outcome of interest. Patients were censored at the occurrence of a competing event or at the last date of observation. The cHRs for the secondary outcomes, i.e. second LRR, SPBC, or death, were shown to give a complete overview of the association between way of LRR-detection and risk of a DM. 2. Too little events occurred to calculate the adjusted cHR | | | | | |

Supplementary Table 4 Cause-specific hazard ratios and 95% confidence intervals for the association between symptoms (yes or no) with risk of development of a distant metastasis (n=107)^a^

|  | Distant metastasis | | Second locoregional recurrence^b^ | Second primary cancer^b^ | Death^b^ |
| --- | --- | --- | --- | --- | --- |
|  | Crude  HR (95% CI) | Adjusted  HR (95% CI) | Crude  HR (95% CI) | Crude  HR (95% CI) | Crude  HR (95% CI) |
| Symptoms |  |  |  |  |  |
| No | 1.00 (reference) | 1.00 (reference) | 1.00 (reference) | 1.00 (reference) | 1.00 (reference) |
| Yes | 1.77 (0.77 – 4.07) | 1.46 (0.51 – 4.20) | 0.97 (0.26 – 3.62) | 0.49 (0.08 – 2.94) | 0.73 (0.21 – 2.54) |
| Tumor size |  |  |  |  |  |
| <2 cm | 1.00 (reference) | 1.00 (reference) | 1.00 (reference) | 1.00 (reference) | 1.00 (reference) |
| 2–5 cm | 2.37 (1.01 – 5.56) | 1.40 (0.50 – 3.87) | 1.30 (0.32 – 5.22) | 1.10 (0.15 – 8.17) | 1.20 (0.34 – 4.22) |
| >5 cm | 10.77 (2.23 – 52.07) | 16.62 (1.65 – 167.53) | 8.59 (0.93 – 79.13) | - | - |
| Grade |  |  |  |  |  |
| 1 | 1.00 (reference) | 1.00 (reference) | 1.00 (reference) | 1.00 (reference) | 1.00 (reference) |
| 2 | 0.97 (0.25 – 3.71) | 0.90 (0.16 – 5.00) | - | 1.07 (0.11 – 10.35) | 2.33 (0.28 – 19.62) |
| 3 | 2.95 (0.83 – 10.42) | 2.37 (0.27 – 20.85) | - | 0.51 (0.03 – 8.30) | 1.86 (0.19 – 18.10) |
| Positive nodes |  |  |  |  |  |
| 0 | 1.00 (reference) | 1.00 (reference) | 1.00 (reference) | 1.00 (reference) | 1.00 (reference) |
| 1–3 | 1.11 (0.48 – 2.60) | 0.54 (0.19 – 1.57) | 2.17 (0.54 – 8.68) | 0.57 (0.06 – 5.66) | 2.31 (0.66 – 8.05) |
| >3 | 3.01 (0.68 – 13.26) | 2.60 (0.48 – 14.17) | 7.12 (0.76 – 66.56) | 10.44 (0.94 – 115.75) | - |
| Type of surgery |  |  |  |  |  |
| Breast conserving | 1.00 (reference) | 1.00 (reference) | 1.00 (reference) | 1.00 (reference) | 1.00 (reference) |
| Mastectomy | 4.36 (1.64 – 11.56) | 3.72 (1.16 – 11.96) | 1.99 (0.50 – 7.98) | 0.47 (0.07 – 3.10) | 1.51 (0.42 – 5.52) |
| Grade of the LRR |  |  |  |  |  |
| 1 | 1.00 (reference) | 1.00 (reference) | 1.00 (reference) | 1.00 (reference) | 1.00 (reference) |
| 2 | 1.60 (0.25 – 10.21) | 1.23 (0.13 – 11.39) | - | - | 0.73 (0.10 – 5.52) |
| 3 | 1.99 (0.32 – 12.27) | 1.22 (0.08 – 18.17) | - | - | 0.48 (0.04 – 5.69) |
| Positive nodes of the LRR |  |  |  |  |  |
| 0 | 1.00 (reference) | 1.00 (reference) | 1.00 (reference) | 1.00 (reference) | 1.00 (reference) |
| 1–3 | 2.91 (1.01 – 8.45) | 1.97 (0.47 – 8.16) | 4.77 (0.85 – 26.91) | 6.71 (0.78 – 59.77) | 1.13 (0.14 – 9.21) |
| >3 | 2.35 (0.79 – 6.98) | 1.09 (0.26 – 4.61) | 4.52 (0.78 – 26.31) | - | - |
| CI: confidence interval, cHR: cause-specific hazard ratio, DM: distant metastasis, LRR: locoregional recurrence, SPBC: second primary breast cancer.   1. The development of a DM, second LRR, SPBC, or death was regarded as a competing event, when not regarded as the outcome of interest. Patients were censored at the occurrence of a competing event or at the last date of observation. The cHRs for the secondary outcomes, i.e. second LRR, SPBC, or death, were shown to give a complete overview of the association between way of LRR-detection and risk of a DM. 2. Too little events occurred to calculate the adjusted cHR | | | | | |
